# Improved Visualization of Intracranial Vessels with Intraoperative Coregistration of Rotational Digital Subtraction Angiography and Intraoperative 3D Ultrasound

**DOI:** 10.1371/journal.pone.0121345

**Published:** 2015-03-24

**Authors:** Dino Podlesek, Tobias Meyer, Ute Morgenstern, Gabriele Schackert, Matthias Kirsch

**Affiliations:** 1 Department of Neurosurgery, Dresden University of Technology, Carl Gustav Carus Faculty of Medicine, Dresden, Germany; 2 Institute of Biomedical Engineering, Dresden University of Technology, Faculty of Electrical Engineering and Information Technology, Dresden, Germany; Heinrich-Heine University, GERMANY

## Abstract

**Introduction:**

Ultrasound can visualize and update the vessel status in real time during cerebral vascular surgery. We studied the depiction of parent vessels and aneurysms with a high-resolution 3D intraoperative ultrasound imaging system during aneurysm clipping using rotational digital subtraction angiography as a reference.

**Methods:**

We analyzed 3D intraoperative ultrasound in 39 patients with cerebral aneurysms to visualize the aneurysm intraoperatively and the nearby vascular tree before and after clipping. Simultaneous coregistration of preoperative subtraction angiography data with 3D intraoperative ultrasound was performed to verify the anatomical assignment.

**Results:**

Intraoperative ultrasound detected 35 of 43 aneurysms (81%) in 39 patients. Thirty-nine intraoperative ultrasound measurements were matched with rotational digital subtraction angiography and were successfully reconstructed during the procedure. In 7 patients, the aneurysm was partially visualized by 3D-ioUS or was not in field of view. Post-clipping intraoperative ultrasound was obtained in 26 and successfully reconstructed in 18 patients (69%) despite clip related artefacts. The overlap between 3D-ioUS aneurysm volume and preoperative rDSA aneurysm volume resulted in a mean accuracy of 0.71 (Dice coefficient).

**Conclusions:**

Intraoperative coregistration of 3D intraoperative ultrasound data with preoperative rotational digital subtraction angiography is possible with high accuracy. It allows the immediate visualization of vessels beyond the microscopic field, as well as parallel assessment of blood velocity, aneurysm and vascular tree configuration. Although spatial resolution is lower than for standard angiography, the method provides an excellent vascular overview, advantageous interpretation of 3D-ioUS and immediate intraoperative feedback of the vascular status. A prerequisite for understanding vascular intraoperative ultrasound is image quality and a successful match with preoperative rotational digital subtraction angiography.

## Introduction

Preoperative investigations of intracranial aneurysms are: Computed tomography angiography (CTA) magnetic resonance imaging angiography (MRA) and digital subtraction angiography (DSA). According to our experience, the interpretation of preoperative diagnostic imaging does not always match the real intraoperative situation in patients.

Complete clipping of intracranial aneurysms, preservation of the vascular tree, and identification of vessels beyond the visual field are the primary goals during aneurysm surgery. Le Roux et al. detected aneurysm remnants in 5.7% of 637 patients treated by clipping upon postoperative angiography [1]. Mere use of a microscope for aneurysm surgery is limited to the visual field. The intraoperative assessment of aneurysm clip placement has been evaluated by intravenous fluorescence angiography and intraoperative near-infrared indocyanin green video angiography [2–4]. As these techniques are limited to the visual field, possible entrapment and affection of distal vessels, e.g. by improper clip placement, may be missed. Intraoperative ultrasound (3D-ioUS) might improve this uncertainty since it is not limited to the visual field and allows for online imaging and real time update of images, respectively. Previous 3D-ioUS reports have shown that 3D-ioUS identified up to 90% of cerebral aneurysms using 2-dimensional transcranial color-coded ultrasonography [5,6]. In addition, advanced 3D-ioUS can render reconstructions of the vascular tree within seconds, therefore offering an additional measure to improve the diagnostic reconstruction of the vessel-tree and, thus, intraoperative safety [7].

The purpose of our study was to evaluate the usability, the accuracy and the intraoperative applicability of volume-based registration of 3D-ioUS in comparison with preoperative rDSA

## Patients and Methods

### Patients

Thirty-nine patients (13 men, 26 women, median age 57 years, range 35–80 years) with 59 saccular aneurysms were included in this study. Forty-three of 59 aneurysms were chosen for neurosurgical clipping and intraoperative ultrasound imaging. The rest of the aneurysms were treated with coiling. Twenty-one patients underwent elective procedures and 18 were treated within 24 hours after suffering from subarachnoid hemorrhage (SAH; Hunt & Hess grades II-V).

### Data acquisition

Our study was performed according to the Declaration of Helsinki on Biomedical Research involving Human Subjects. It was approved by the Ethics Committee of the Faculty of Medicine Carl Gustav Carus at the Technische Universität Dresden, Germany (EK63042005; EK348092013). The participants or their relatives provided informed consent either written or verbally to participate in this study. In six patients, written consent was not obtained but approval was given by their relatives and documented in the patients file.

### Pre-operative imaging studies

Thirty-nine patients underwent DSA with 3D. Selective pan-angiography DSA scans with multiple planes of each carotid artery and 4 planes of vertebrobasilar tree were routinely performed. In 6 patients, preoperative computed tomographic angiography (CTA) was obtained for the acute diagnostic approach.

### Clipping procedure

The head was fixed in a Sugita head frame. Pterional craniotomy or modified frontotemporal approach were chosen for clipping the aneurysms located on anterior cerebral circuit. Before aneurysm dissection, the ultrasound volume was obtained. This was accomplished by holding the ultrasound probe manually or by immobilizing the probe with a pneumatic-flexible arm (Unitrack Aesculap, Tuttlingen, Germany). Setup and completion time were less than 10 minutes, even if several volumes were acquired. The time limiting factor was most frequently a technical issue, human error, or limitation due to a poor clinical status of the patient.

Consecutively, 3D-ioUS data sets were imported to a laptop with a stereoscopic display and coregistered with preoperative rDSA resulting in a rapid 3D display of the coregistered vascular tree. Routinely, intraoperative micro-Doppler measurements were performed before and after clip placement using a 16 MHz conventional micro-Doppler probe (diameter 1 mm, DWL, Singen Germany) by applying the ultrasound-probe directly on the visible vessels. In selected patients, near-infrared indocyanine green (ICG) videoangiography was performed as well [3].

### Intraoperative 3D-ultrasound

Intraoperative high-resolution power Doppler 3D imaging (Voluson 730, GE Healthcare, formerly Kretz, Brussels) was used in 39 patients with anterior, middle cerebral and internal carotid artery aneurysms to visualize the aneurysm and the vascular tree prior to and after clipping. For this purpose, a curved linear array transducer (4D micro-convex endocavitary transducer, 5–9 MHz ultrasound probe, RIC5–9 curved linear array volumetric real time 3D probe, GE Healthcare, formerly Kretz, Brussels) was used. The ultrasound probe contains the mechanism that pivots the ultrasound crystals during the acquisition of volume data. The probe was attached to a pneumatic arm (Unitrack Aesculap, Tuttlingen, Germany) to minimize motion artefacts. Both power Doppler and conventional B-mode volume data sets were captured in varying B-image angles of 40°-130° and panning angles of 20°-95°. Before the coregistration procedure and data reconstruction of the intracranial vessels, analysis of 3D-ioUS MIP (maximum intensity projection; MIP) images was performed. Whenever possible, series of 3D power Doppler and color-coded duplex measurements were performed. These data were transferred to a laptop with a 3D display within eye-sight of the neurosurgeon.

### Hardware and software visualization tools

A notebook with integrated autostereoscopic display (Sharp AL3DU; Sharp Corporation, Osaka, Japan) enabled immediate 3D impressions of the vascular morphology without using additional viewing aids such as shutter glasses.

A 3D mouse (Spaceball 5000, 3DConnexion, Fremont, California, USA) supported navigating and maneuvering objects in 3D space. Digital 3D planning and editing was performed using the commercial visualization software package AMIRA (Visage Imaging, Berlin, Germany, formerly Mercury Computer Systems, TGS Inc., San Diego, USA). Beyond basic 3D software tools, AMIRA approves development and integration of customized 3D visualization and processing algorithms. Hence, specific software modules were implemented for neurosurgical-oriented demand. The 3D-ioUS steps of processing and visualization have been automated and the interactive steps have been reduced to a minimum during the process of coregistration and visualization. 3D-reconstruction, visualization and coregistration of rDSA, as well as 3D-ioUS were immediately performed after ultrasound imaging. US data sets were post processed by digital elimination of artefacts (isolated voxel), visual optimization (smoothing with Gaussian filter), and updating those for intraoperative rDSA fusion. An automated rigid (translation, rotation) registration algorithm (standard AMIRA module AffineRegistration) registered the 3D-ioUS data set to the reference data set (rDSA). The algorithm progressed stepwise for multiple resolutions: Registration is initially performed with a coarse resolution of the data sets. Then, a registration with finer resolutions used the transformation parameters of the prior registration. Due to the different modalities of the image data, the normalized mutual information metric was used to estimate the similarity between rDSA and 3D-ioUS for automatic registration. In case of insufficient alignment, the registration was manually conducted using the 3D navigation device.

For a more accurate comparison of 3D-ioUS data with preoperative rDSA, local misalignment was compensated. These misalignments originated from deformation of the brain because of cranial opening and the different imaging geometry of rDSA and 3D-ioUS. A non-rigid registration algorithm can compensate for local deformations of the 3D-ioUS data after rigid registration. The module was programmed using the B-Spline deformable transform from the Insight Segmentation and Registration Toolkit (ITK) [8]. An evenly spaced grid was laid over the 3D-ioUS data set. The grid nodes can move in each dimension independently. Parameterized cubic curves (B-Splines) interpolated the deformation between the single grid nodes. The normalized mutual information was used as similarity metric. The grid nodes moved in respect to the optimization of the similarity between the data sets. After coregistration, the rDSA and the 3D-ioUS were visualized. A surface rendering algorithm (standard AMIRA module IsoSurface) visualized the rDSA data sets. The surface of the vessels were reconstructed as a tetrahedral grid and visualized as a shaded surface. 3D-volume rendering visualized the 3D-ioUS data set. Transparent volume rendering made simultaneous perceptibility of both data sets possible.

All data processing steps described above were integrated into a single AMIRA-based program which was executed on demand. For practicability and ease of 3D navigation, a 3D mouse encased into a sterile wrapping was available on demand for the neurosurgeon. All patients with ultrasound data sets were evaluated retrospectively as well.

### Post-operative imaging

All patients had a postoperative CT scan on the following day. For detection of vasospasm, Transcranial Doppler US of intracranial vessels (DWL Doppler; Compumedics GmbH, 2MHz probe, Singen, Germany) was performed daily to bidaily. Within one week, postoperative conventional digital subtraction angiography was performed in all patients. In 6 patients, postoperative rDSA was performed because the patients had a second surgery for an additional aneurysm or unclear remnants in conventional DSA. To validate intraoperative findings, post-operative control rDSA results were matched with the intraoperatively acquired 3D-ioUS volume.

### Assessment of 3D-ioUS quality

We determined the intraoperative aneurysm carrier morphology, aneurysm volume, aneurysm neck width, presence of vasospasm, and compared those to preoperative rDSA. 3D-ioUS data were evaluated by an ultrasound experienced vascular neurosurgeon. Postoperatively, those were re-evaluated independently by two neurosurgeons with regard to aneurysm visualization and anatomy, detection of parent vessel, aneurysm neck depiction, and successful coregistration of 3D-ioUS and rDSA. Post-processing and intraoperative adaption of US data sets were time consuming, which improved over time, resulting in a data acquisition and processing time of less than 15 min.

### Similarity of aneurysm volume visualization of rDSA and 3D-ioUS

**Fig 1:**
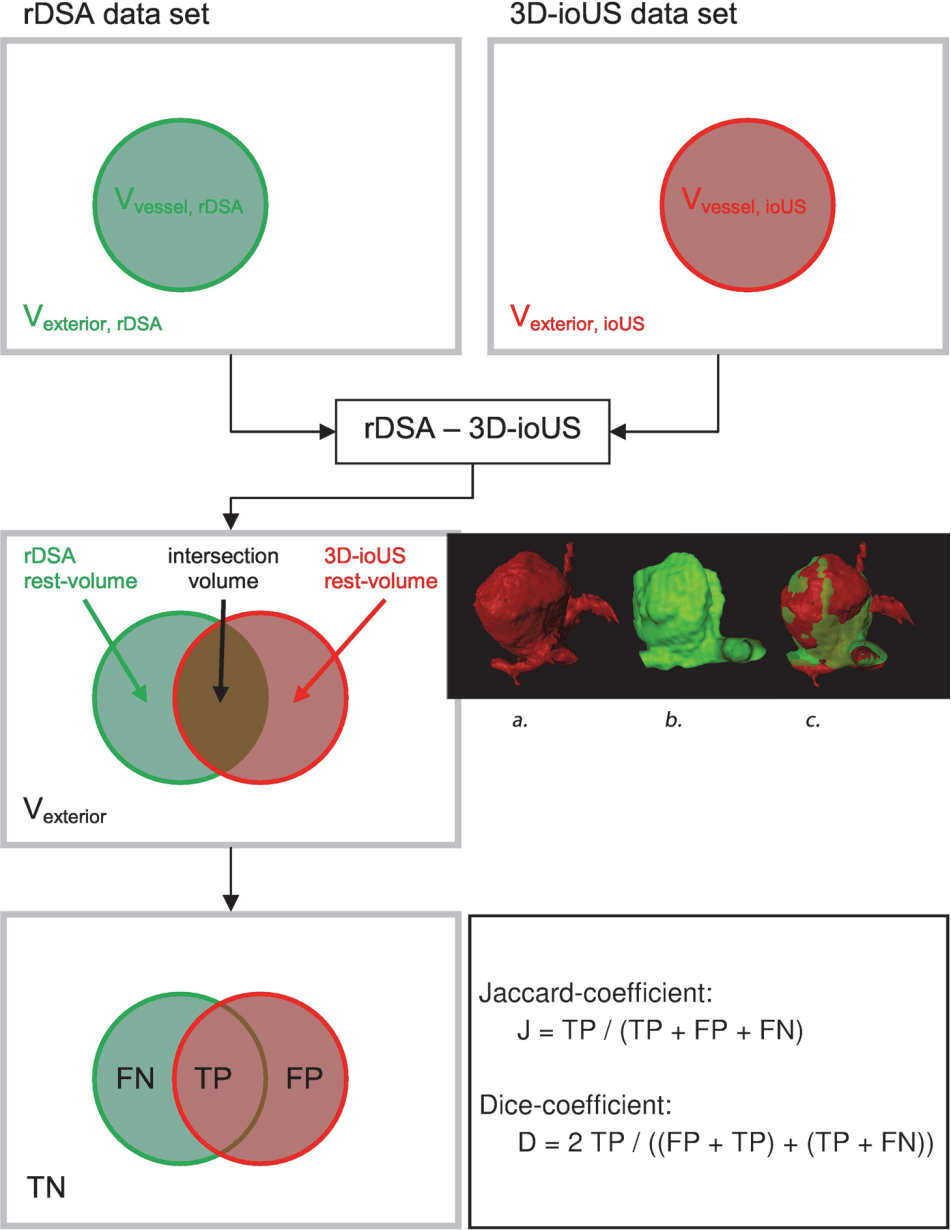
Pattern of the evaluation of registration results by overlapping the rDSA and the 3D-ioUS (rDSA rotational digital subtraction angiography, 3D-ioUS: intraoperative 3D-ultrasound, TN: true negative, FN: false negative, TP: true positive, FP: false positive). Evaluation of registration; a.: preoperative rDSA; b.: preoperative 3D-ioUS; c.: intraoperative coregistration of a. and b.

We compared the volume (voxel count) overlap between the rDSA (Vvessel, rDSA, reference data set) and the 3D-ioUS (Vvessel, ioUS, transformed data set) to evaluate the similarity after registration (Fig. 1). The volume of interest (VOI) was centered on the aneurysm in both data sets and cropped to fit the aneurysm including the aneurysm neck. The true positive (TP), the false positive (FP), and the false negative (FN) counts of the overlap were calculated for each registration. The TP value describes the intersection between the rDSA and the transformed 3D-ioUS. FN and FP represent the non-overlapping volumes of the DSA and the 3D-ioUS, respectively.

These data were used to calculate both the Jaccard and the Dice coefficients [9]. The Jaccard- index describes the size of the intersection of the volume of the two data sets relative to the size of their union (J(A,B) = (A and B)/(A or B)). Dice-coefficient displays more robust estimation of spatial overlap than the Jaccard-index, using the following equation: D(A,B) = 2 (A and B)/(A + B) where A and B are the vessels in the rDSA data set and the 3D- ioUS data sets, respectively. This formula represents the size of the union of 2 sets divided by the average size of the both sets. It can be expressed as: D(A,B) = 2 TP /((FP+TP)+(TP+FN)). It equals 1 if the volumes overlap is complete and equals 0 if there is no overlap (Fig. 1).

## Results

### Patient characteristics

Before the aneurysm dissection and clipping, we obtained 3D-ioUS volumes in 39 patients and repeated the measurement in 26 patients after clipping. Thirty-nine patients harbored 43 aneurysms and underwent both 3D-ioUS and rDSA. Duplex sonography of associated intracranial vessels was obtained in 24 patients.

As a result of improper insonation in 5 out of 39 patients (13%), aneurysms were outside of the field of view, yet they provided the additional information on ipsilateral vascular tree beyond the visual field. We visualized 35 of 43 aneurysms (81%) with 3D-ioUS and reconstructed those successfully with AMIRA software. Due to inaccurate insonation angle and aneurysm wall calcification in patient 13 and patient 32 only the aneurysm neck and the outgoing vessels were visualized and successfully matched.

Simultaneous coregistration of preoperative rDSA data with intraoperative pre-clipping 3D-ioUS was performed sufficiently in 37 of 39 (95%) patients. The coregistration was insufficient in 8 of 26 patients in post clipping 3D-ioUS due to clip artefacts.

The result of coregistration was demonstrated to two ultrasound-experienced vascular neurosurgeons who subjectively evaluated the coregistration results intraoperatively. Their assessment was excellent with coregistered identification of the vascular structures (Table 1).

**Table 1:** Evaluation of 3D-ioUS.

Patient	Location	Aneurysm volume (cm3)	3D-ioUS availability	3D-ioUS sufficiency	Aneurysm	Carrier vessel	Aneurysm neck	Outgoing vessels	Coregistration of ioUS/rDSA	Comments
			Pre / post Clip							
1	MCA	0.133	+/−	+++/−	+++/−	+++/−	+++/−	+++/−	+/−	
2	MCA	0.298	+/+	+++/+++	+++/+++	+++/+++	+++/++	+++/++	+/+	
3	MCA	0.08	+/−	+++/−	+++/−	+++/−	+++/−	+++/−	+/−	
4	MCA	0.044	+/+	+++/+++	+++/++	+++/++	+++/+++	+++/+++	+/+	
5	MCA	1.44	+/−	+++/−	+++/−	+++/−	+++/−	++/−	+/−	
*6*	*AcommA*	1.4	*+/−*	*+/−*	*−/−*	*−/−*	*−/−*	*+/−*	*−/−*	*Aneurysm not in FOV*
7	MCA/ +ICA-T	0.03	+/+	+++/+++	+++/+++	+++/+++	++/++	+++/+	+/+	
*8*	*AcommA*.	0.22	*+/−*	*++/−*	*−/−*	*−/−*	*−/−*	*−/−*	*+/−*	*Aneurysm not in FOV*
*9*	*AcommA*.	0.16	*+/−*	*+++/−*	*−/−*	*−/−*	*−/−*	*−/−*	*+/−*	*Aneurysm not in FOV*
10	MCA	0.76	+/−	+++/−	++/−	++/−	++/−	++/−	+/−	
*11*	*AcommA*.	0.98	*+/−*	*++/−*	*−/−*	*−/−*	*−/−*	*−/−*	*+/−*	*Aneurysm not in FOV*
12	AcommA.	1.17	+/+	+++/+	+++/−	+++/−	+++/++	++/−	+/+	
*13*	*AcommA*.	0.64	*+/+*	*+++/+*	*−/−*	*+++/+*	*++/−*	*+++/−*	*+/−*	*Thrombosed aneurysm*
14	MCA	1.32	+/+	+++/+	+++/−	+++/−	+++/−	++/−	+/−	
15	MCA	0.084	+/+	+++/+++	+++/++	+++/++	+++/+++	+++/++	+/+	
16	MCA	0.21	+/−	+++/−	++/−	+++/−	++/−	+++/−	+/−	
17	MCA	0.81	+/+	+++/+	+++/−	++/+	++/−	+++/+	+/−	
18	MCA	0.13	+/+	++/+	++/−	+/−	+/−	++/++	+/+	
19	MCA/MCA	0.036	+/+	+++/+++	++/++	+++/+++	+++/+++	+++/+++	+/+	
20	MCA	0.36	+/+	+++/+++	+++/++	+++/++	+++/++	++/++	+/+	
21	MCA/MCA	0.165	+/+	+++/++	+++/+	+++/+	+++/+++	+++/++	+/+	
22	MCA/MCA/MCA	0.364	+/+	+/+	+/+	+++/++	++/++	++/++	+/+	
23	MCA	0.01	+/−	+++/−	++/−	++/−	++/−	++/−	+/−	
24	MCA/MCA	0.7	+/+	+++/+	+++/−	+++/+	++/−	++/−	+/−	
25	MCA	0.247	+/+	+++/++	++/++	+++/++	++/+++	+++/++	+/+	
*26*	*AcommA*./*MCA*	0.04	*+/+*	*+/+*	*−/−*	*+/−*	*+/−*	*+/−*	*−/−*	*Aneurysm not in FOV*
27	MCA	0.56	+/+	+++/+++	+++/+++	+++/+++	+++/+++	+++/+++	+/+	
28	AcommA./MCA	0.193	+/+	+++/++	++/++	+++/+++	+++/+++	++/++	+/+	
29	MCA	2.02	+/+	++/+	++/−	++/−	++/−	++/−	+/−	
30	MCA/MCA/MCA	0.59	+/−	+++/−	+++/−	+++/−	+++/−	+++/−	+/−	
31	AcommA	0.77	+/+	+++/+	+++/−	+++/−	+++/−	++/−	+/−	
*32*	*ICA-T*	1.1	*+/−*	*++/−*	*++/−*	*++/−*	*++/−*	*++/−*	*+/−*	*Aneurysm partially thrombosed*
33	MCA	2.5	+/−	+++/−	+++/−	+++/−	+++/−	++/−	+/−	
34	MCA	0.068	+/−	+++/−	+++/−	+++/−	+++/+++	−/−	+/−	
35	ICA-T	1.81	+/−	++/−	++/−	++/−	++/−	++/−	+/−	
36	MCA	0.81	+/+	+++/−	+++/−	+++/−	+++/−	+++/+	+/−	
37	AcommA/MCA/MCA	0.14	+/−	+++/−	+++/−	+++/−	++/−	++/−	+/−	
38	AcommA	0.1	+/+	+/−	+/−	−/−	−/−	−/−	−/−	
39	MCA	0.035	+/+	+++/++	+++/++	++/++	++/++	++/+++	+/+	

Aneurysm volumes were calculated from the maximum intensity projection (MIP) volumes of ioUS. As indicated, ioUS measurements were performed pre- and post-clipping. Two independent investigators evaluated the data which is depicted on a scale from – (not available or insufficient images) to +, ++, or +++. The overlap presents the volumetric ratio of the aneurysms.

### Accuracy of rDSA—3D-ioUS coregistration

We used the Dice-coefficient and Jaccard-index to quantify the intersection overlap between 3D-ioUS aneurysm volume and preoperative rDSA aneurysm volume (Table 2). The segmented volumes of 3D-ioUS and preoperative rDSA data sets were very similar with 94% accuracy. Although the aneurysm volumes were approximately the same size in rDSA and 3D-ioUS, they had slightly different 3D conformations resulting in a Jaccard-index of 0.56. The brain shift could be one of the factors influencing those data. Therefore, the Dice-coefficient was employed to provide a more robust estimate [9] of the spatial similarity of different imaging modalities resulting in a good overlap of 0.71. This should provide a clinically meaningful index to assess the comparability of the imaging modalities.

**Table 2:** Results of Dice-coefficient, Jaccard-Index and volume ratios for rDSA and 3D-ioUS volumes. (Due to incomplete aneurysm visualization or absent aneurysm in the field of view patients 6, 8, 9, 11, 13, 26 and 32 were excluded from calculations).

Patient	Aneurysm	Jaccard-index	Dice-coefficient	OverlapV_3D-ioUS_: V_rDSA_
1	*MCA*	0.76	0.87	0.93
2	*MCA*	0.58	0.74	0.77
3	*MCA*	0.59	0.74	0.68
4	*MCA*	0.47	0.64	0.76
5	MCA	0.45	0.62	0.86
6	AcommA	-	-	-
7	*MCA*	0.27	0.43	0.89
8	*AcommA*	-	-	-
9	*AcommA*	-	-	-
10	*MCA*	0.74	0.85	0.96
11	*AcommA*	-	-	-
12	*AcommA*.	0.43	0.60	0.93
13	*AcommA*	-	-	-
14	*MCA*	0.22	0.36	0.80
15	*MCA*	0.61	0.76	1.20
16	*MCA*	0.59	0.74	0.99
17	*MCA*	0.52	0.68	0.97
18	*MCA*	0.51	0.68	0.89
19	*MCA*	0.56	0.72	0.84
20	*MCA*	0.46	0.63	0.89
21	*MCA*	0.66	0.80	0.97
22	*MCA*	0.61	0.76	0.97
23	*MCA*	0.65	0.79	0.83
24	*MCA*	0.52	0.69	0.90
25	*MCA*	0.71	0.83	1.01
26	*AcommA*./*MCA*	-	-	-
27	*MCA*	0.70	0.82	1.06
28	*AcommA*.	0.61	0.76	1.08
29	*MCA*	0.63	0.78	1.15
30	*MCA*	0.54	0.70	1.13
31	*AcommA*	0.68	0.81	1.18
32	*ICA-T*	-	-	-
33	*MCA*	0.56	0.72	1.04
34	*MCA*	0.56	0.72	0.85
35	*ICA-T*	0.52	0.69	0.94
36	*MCA*	0.66	0.79	1.01
37	*AcommA*	0.50	0.67	1.02
38	*AcommA*	0.40	0.58	0.88
39	*MCA*	0.60	0.75	1.05
Mean		0.56	0.71	0.94

The index equals 1 if the volumes overlap completely and 0 if there is no overlap.

### Coregistration of preoperative 3D-ioUS and rDSA

#### Illustrative cases

In patient 13, the aneurysm was only partially visualized in 3D-ioUS compared to rDSA, suggesting either a partially thrombosed aneurysm or a thickened and calcified aneurysm wall.

**Fig 2:**
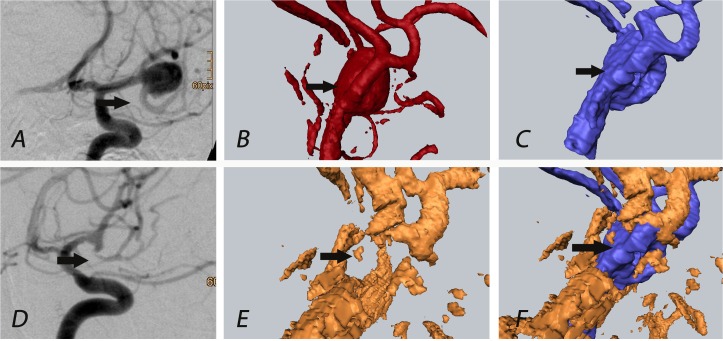
Patient 17: The black arrow indicates M2 segment (temporal branch of MCA) of the medial cerebral artery, vasospasm of M2 segment visualized in post-clipping 3D-ioUS (E) which led to repositioning of the clip; (rDSA (red), 3D-ioUS (blue, orange); A: pre-clipping conventional DSA; B: pre-clipping rDSA; C: pre-clipping 3D-ioUS; D: post-clipping conventional DSA; E: post-clipping 3D-ioUS; F: coregistration of C+E.

In patient 17, a complex MCA bifurcation aneurysm was clipped. 3D-ioUS obtained a good match with the pre-operative rDSA, however, it showed a vasospasm of the temporal M2 branch distal to the clip. This finding was confirmed in postoperative conventional DSA and in the corresponding MIP images of 3D-ioUS, although this was initially interpreted as an occlusion of M2-segment in 3D-ioUS (Fig. 2).

**Fig 3:**
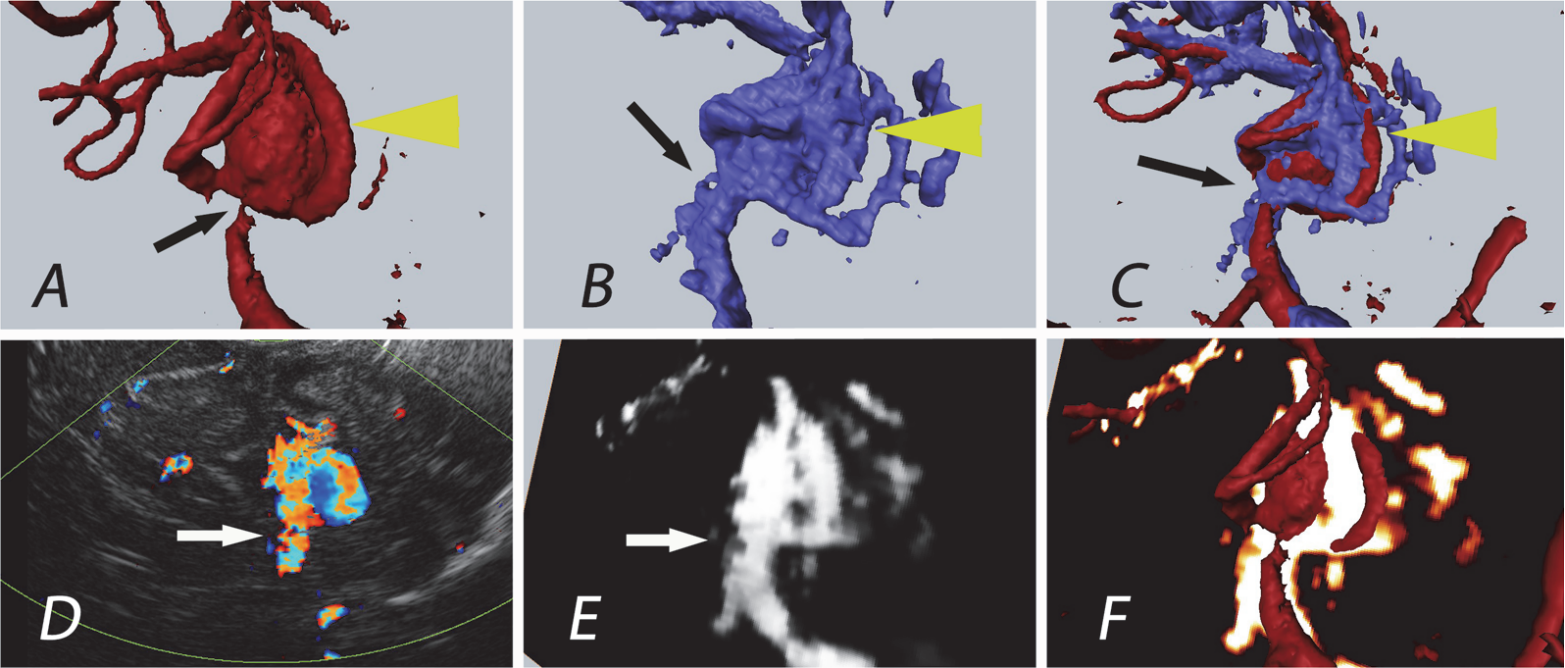
Patient 5: The yellow arrow indicates the aneurysm of the medial cerebral artery bifurcation; the black Arrow indicates the aneurysm origin/neck; improved visualization of the aneurysm neck in 3D-ioUS (B) with narrow M1 segment of the medial cerebral artery due to high blood flow velocity in bifurcation marked with a white arrow (D, E).; rDSA (red), 3D-ioUS (blue, glow-orange), A: pre-clipping rDSA; B: pre-clipping 3D-ioUS; C: coregistration of A+B; D: pre-clipping duplex; E: MIP 3D-ioUS; F: coregistration of A+E.

In patients 5, 29, and 30, the combination of 3D-ioUS with rDSA revealed details not apparent on the single modality imaging. The combination with ultrasound flow measurements (duplex) demonstrated a degree of detail that was not visible in rDSA alone. This is potentially an effect of high flow velocity in the parent vessel. In patient 5, 3D-ioUS provided additional information of M1 branch anatomy (Fig. 3).

**Fig 4:**
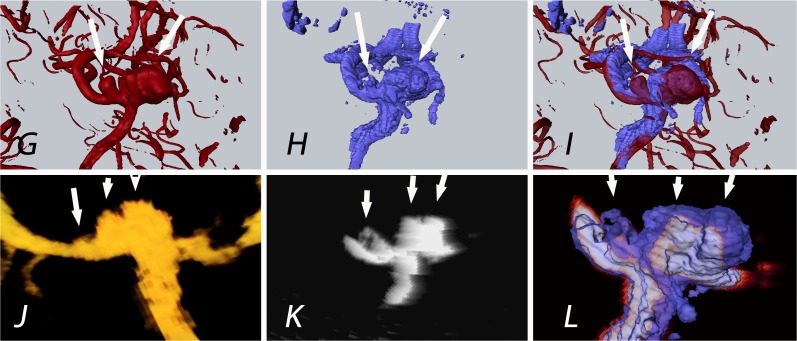
Patient 30: The white arrows indicate the aneurysms of the medial cerebral artery bifurcation. Contrary to rDSA (G; 2 aneurysms detected), 3 aneurysms were displayed in 3D-ioUS (H, J, K; 2 on MCA bifurcation and the 3rd aneurysm on M2 branch) as confirmed by the intraoperative view; (rDSA (red), 3D-ioUS (blue, glow- orange)); G: pre-clipping rDSA; H: pre-clipping 3D-ioUS; I: coregistration of G+H; J: MIP 3D-ioUS; K: MIP of 3D-ioUS; L: coregistration of K+H.

In particular, the aneurysm neck was clearly separated from the surrounding artery. In patient 30, 3D-ioUS demonstrated two separate aneurysm domes in contrast to a fused dome in pre-operative rDSA (Fig. 4).

**Fig 5:**
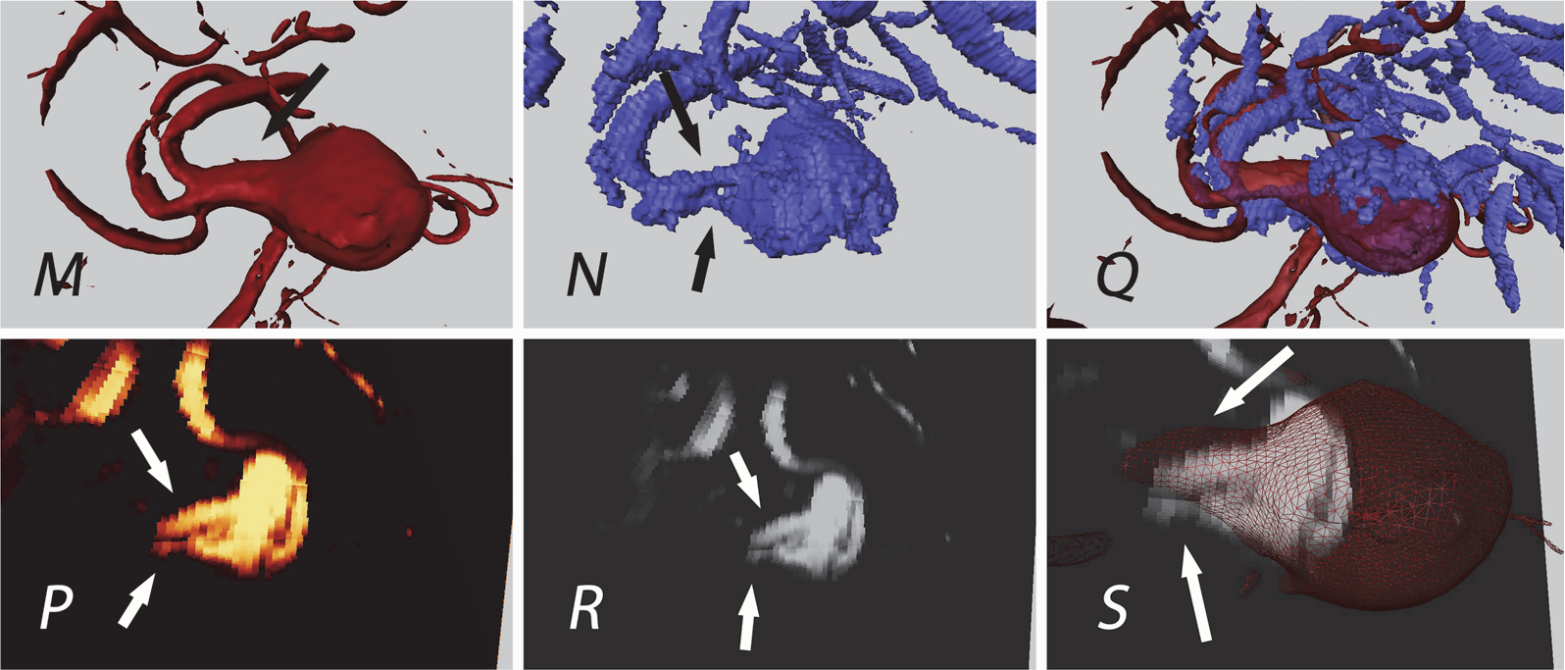
Patient 29: Aneurysm of the medial cerebral artery bifurcation; the black and white arrows indicate the M2 segments of the medial cerebral artery; uncertainty in preoperative rDSA interpretation of M2 segment (single broad vessel vs. 2 nearby vessels); detection of 2 M2 branches (N, P and R) in 3D-ioUS as confirmed intraoperatively; rDSA (red), 3D-ioUS (blue, glow-orange); M: pre-clipping rDSA; N: pre-clipping 3D-ioUS; O: coregistration of M+N; P,Q: MIP of 3D-ioUS; R: coregistration of R+M.

Similarly, patient 29 revealed a better separation of individual M2 branches compared to rDSA (Fig. 5).

3D-ioUS had its limitations due to calcification and clip artefacts. It gave us an assumption of aneurysm calcifications by producing acoustic shadowing. In some patients, acoustic shadowing hindered the interpretation of 3D-ioUS. Several 3D-ioUS volumes of the region of interest were necessary to interprete the vascular anatomy (patients 2, 4, 13, 17, 24, 27, 30, 35). Pre-existing clip placement generated artefacts in patient 24, however, imaging at different angles made the aneurysm visualization possible.

**Fig 6:**
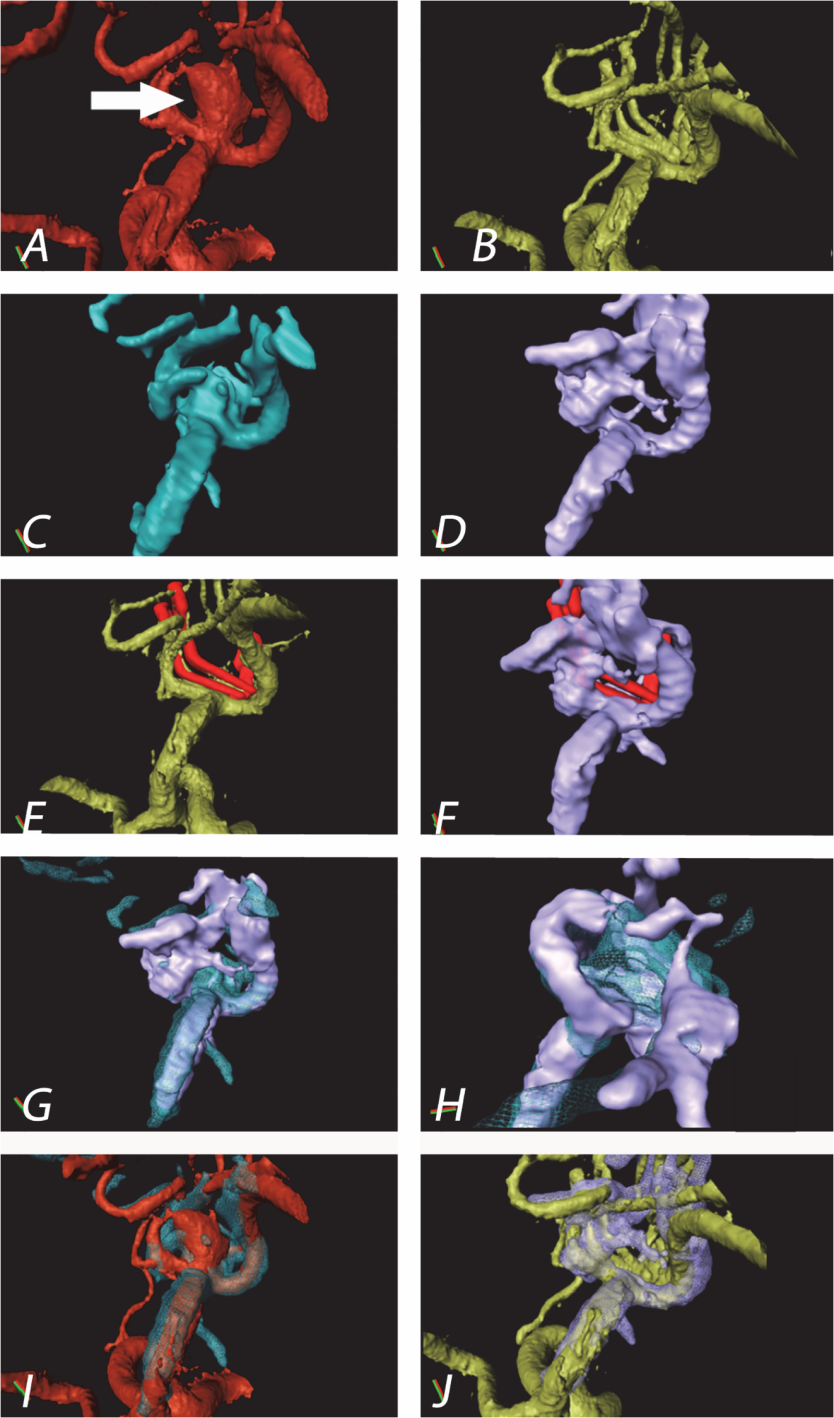
Patient 4: Aneurysm of the medial cerebral artery bifurcation indicated by the white arrow. Precise aneurysm neck visualization in 3D-ioUS and clip segmentation for virtual planning of clipping procedure. **A**: pre-clipping rDSA (red); **B**: post- clipping rDSA (green); **C**: pre-clipping 3D-ioUS (blue); **D**: post-clipping 3D-ioUS (pink); **E**: post- clipping rDSA with segmented clips (red); **F**: post- clipping 3D-ioUS with segmented clips (red); **G—H**: coregistration of C+D; I: coregistration of A+C; **J**: coregistration of B+G.

By obtaining several 3D-ioUS volumes from different angles and fixing the probe to a pneumatic arm, clip and motion artefacts decreased to a minimum. The parent vessel and the aneurysm neck were visualized with high precision in absence of motion artefacts (Fig. 6).

## Discussion

Intraoperative semi-automated coregistration of 3D intraoperative ultrasound with preoperative rDSA combines the advantages of ultrasound, namely speed, non-invasiveness and visualization beyond the visible field, with an improved anatomical interpretation of the coregistered 3D-US images. Using standard visualization tools, it is possible to simulate and evaluate the intraoperative strategy when the anatomical structures were afflicted by brain shifting and other changes concerning the anatomical discrepancy to the preoperative data.

Successful detection and interpretation of ioUS requires a learning curve in particular for small intracranial aneurysms and small vessels. Despite the inability to visualize small neck remnants and perforating vessels after clipping, intraoperative 3D-ioUS coregistered with preoperative rDSA was positively evaluated by independent neurosurgeons.

Since the first report of transcranial ultrasound application, Stendel et al. has shown the reliability, practicability and the necessity of intraoperative microvascular Doppler ultrasonography in cerebral aneurysm surgery [10,11]. The aneurysm detection by transcranial power Doppler depends on aneurysm location and its morphology. In 3D transcranial power sonography, the differentiation of artefacts and real time vascular changes is superior to 2-dimensional transcranial color- coded sonography [5,12]. A diameter smaller than 5–6 mm, poor bone window, and adverse aneurysm location were the cause of insufficient aneurysm visualization using transcranial color-coded duplex sonography [12,13]. Turner et al. showed that the sensitivity and specificity increase with the aneurysm diameter and are 100% in aneurysms with a 12mm diameter. The application of the intravenous contrast agent increases its sensitivity and specificity [14]. Klötzsch et al. reported a higher rate of undetected aneurysms using nonenhanced 3D-TCCS than contrast enhanced ultrasound [12]. Other studies employing neurosonography integrated in neuronavigation systems have been introduced and have clearly shown intraoperative advantages [15–22]. Lindseth et al. have shown that blood-flow imaging as an independent ultrasound modality and integrated with 3D navigation technology has a high potential for the visualization of flow dynamics of intracranial vascular pathologies [13].

In the retrospective analysis of 180 patients with 205 aneurysm Brinjikji et al. showed the differences in measurements of dome-to-neck ratios between 2D and 3D-rDSA [23]. There is a tendency to overestimate the neck size due to volume averaging in 3D imaging techniques. As we have shown for patient 17 the evaluation of intraoperative 2D-ioUS revealed a severe M2 vasospasm previously interpreted as an occlusion in 3D-ioUS. For accurate imaging interpretation and, therefore, assessment of vessel anatomy, 2D imaging is still crucial. The noninvasiveness and possibility of repeated ioUS measurements from different angles are advantageous for improved imaging interpretation.

As an alternative to the established vascular imaging modalities, such as ICG angiography, vascular US and conventional DSA, a selective catheterization of the exposed ipsilateral superficial temporal artery has been proposed as a rapid intraoperative angiography [24]. Although the procedure is simple and can be repeated during the procedure once the catheter is in place, the preparation of the STA is time-consuming compared to US, its usability for DSA not obvious beforehand, it sacrifices the superficial temporal artery, and radiation exposure is inevitable [24]. The major advantage is its superior resolution and comparability with standard DSA imaging.

Sufficient clipping can be accessed by ICG application. Good visualization depends on the aneurysm location relative to the parent vessel and its morphology. Sharma et al. advocated the intraoperative ICG application as a complementary technique to intraoperative digital subtraction angiography [25]. ICG has a superior resolution to visualize small and perforating arteries as long as they are in the visual field. Even though the technique has a low risk profile and is useful for adequacy of aneurysmal obliteration and patency of perforating vessels, the evaluation of perfused vessels is limited to the visual field and is flow dependent [3,26–33]. Only DSA and US are able to visualize blood flow in the non-visible compartment.

Although the intraoperative DSA has been introduced by several studies as a “gold standard” technique for the detection of aneurysm remnants, the intraoperative risk, the invasive procedure and the cost effectiveness are the main disadvantages. The visualization of the perfusion of the perforating vessels is still not sufficient due to imaging resolution of the intraoperative DSA [34–37].

In our study none of the aneurysm remnants (patient 24, 27, and 33) were detected with ioUS due to the immediate proximity to the surgical clip. However, regrown aneurysms were detectable. Pre-clipping, aneurysms were visualized down to 3 mm in diameter. After the clipping procedure, the aneurysm and the parent vessels were better visualized in 3D-ioUS compared to 2D MIP images as seen in several patients. The Dice-coefficient for automatic comparison between two different imaging modalities proved to be more robust than the Jaccard-index. In fact, the aneurysm volumes were very similar upon 3D-ioUS and rDSA imaging. The interpretation of 3D-ioUS is improved when comparing (coregistration) those with preoperatively obtained rDSA data.

The spatial resolution of vascular ultrasound is inferior to standard 3D-DSA, and within the operative field, inferior to ICG angiography. In particular, very small arteries, such as perforating arteries are difficult to discern. However, the diagnostic accuracy continuously evolves with the application of intraoperative ultrasound contrast enhancing agents and newer technologies [13].

Calcified plaques and the clip-material, producing an acoustic shadow, are the limiting factors for intracranial ultrasound imaging. In such cases, we showed that several ioUS-volumes from different angles were required for successful vascular reconstruction. Image reconstruction and coregistration is able to decrease clip artefacts. Even better results might be possible using elastic fusion algorithms [38–40], or the use of adaptive coregistration algorithms for approximation of the resolution of two different image modalities, as well as vascular ultrasound contrast agents.

Despite its limitations, the combined use of the diagnostic information of DSA, ICG and vascular ultrasound imaging fills most intraoperative diagnostic gaps as complementary tools.

Further developments should combine power 3D-ioUS integrated into a neuronavigation system for identification and reduced misinterpretation of ioUS data due to brain-shift, in perspective allowing for transcranial 3D color coded Doppler and the possibility for blood flow velocity quantification in 3D. In addition, mathematical algorithms have been developed to improve the image quality by reducing the artefacts [41,42]. The immediate intraoperative coregistration of 3D-ioUS and rDSA of cerebral aneurysms conveys one of the many benefits of US in the neurosurgical operating theatre and shows that although the ultrasound data volumes were difficult to interpret on their own, the coregistration to rDSA images is important for interpretation.
